# A Case of Chronic Rupture of Achilles Tendon Managed Using a Combination of Multiple Surgical Techniques

**DOI:** 10.7759/cureus.37171

**Published:** 2023-04-05

**Authors:** Vinod Nair, Swaroop Solunke, Vishal S Patil, Satyam Jawa, Rushikesh Abhyankar

**Affiliations:** 1 Orthopaedics, Dr. D. Y. Patil Medical College, Hospital & Research Centre, Pune, IND; 2 Orthopaedic Surgery, Dr. D. Y. Patil Medical College, Hospital & Research Centre, Pune, IND; 3 Orthopaedics and Traumatology, Dr. D. Y. Patil Medical College, Hospital & Research Centre, Pune, IND

**Keywords:** v-y plasty, krackow suture, chronic rupture, acute rupture, achilles tendon

## Abstract

Achilles tendon rupture is a common injury that occurs due to sudden dorsiflexion of the plantar-flexed foot. Both acute and chronic ruptures are frequently misdiagnosed and mistreated. Acute Achilles tendon rupture commonly occurs in middle-aged individuals (30-40 years). Although several operative procedures are available for Achilles tendon repair, the management of choice remains controversial and debatable.

A 27-year-old male came to our clinic complaining of pain over the left ankle for the last five months. History revealed trauma caused by a heavy metal object five months ago. Physical examination revealed tenderness and swelling over the left heel. Ankle plantar flexion was restricted, and painful and squeeze test was positive.

Magnetic resonance imaging was suggestive of a tear of the Achilles tendon in the left ankle. Surgical management was done with multiple techniques which included flexor hallucis longus tendon graft augmentation, end-to-end suturing (Krackow technique), V-Y plasty, and bioabsorbable suture anchor. Although complications such as scar stiffness and wound gaping are common in such cases, the postoperative outcome was excellent in our case according to the American Orthopedic Foot and Ankle Score.

## Introduction

The Achilles tendon is the largest tendon in the human body, which most frequently undergoes a complete subcutaneous tear [1]. It is formed by the confluence of the soleus and gastrocnemius muscles and is one of the most commonly injured tendons. The common causes of Achilles tendon tears include sports injuries, penetrating trauma, or road traffic injuries [2].

Usually, a chronic Achilles tendon rupture occurs four to six weeks after the initial trauma. On inspection, it is impossible to stand on the toes of the affected side and a gap can be felt between the injured ends. Chronic Achilles tendon ruptures are known to result in problems such as tendon retractions with gaps at the cut ends, scarring, calcification, and collagen deterioration [3,4]. The preferred diagnostic technique for identifying Achilles tendon rupture is magnetic resonance imaging (MRI). End-to-end repair is possible only in cases where the gap between tear ends is 2.5 cm or less, while reconstruction using local tissue and local or free tendon grafts is required for larger defects. The surgery is often complicated by wound breakdown and infections owing to the degeneration and poor vascularization of the tendon stumps, its surrounding tissue, and the overlying skin [5].

The non-operative treatment involves immobilizing the foot in plantar flexion for six to eight weeks with a plaster cast or functional brace; however, this causes the gastro-soleus tendon to lengthen, reducing its strength and increasing the risk of rupture.

The benefits of open repair include lowering the re-rupturing rate to 2-5% and making it convenient for patients who need to recover quickly for a job or athletic endeavors [5]. Achilles tendon ruptures with small gaps of up to 2 cm can typically be directly closed end-to-end. Many have advised augmenting end-to-end repair for acute injuries involving fascia flaps or a nearby tendon.

The augmentation technique described by Lindholm et al. prevents the adhesion of the repaired tendon to the surrounding skin by strengthening the patch with two-sided gastro-soleus fascial flaps that have been turned 180 degrees [6].

Although open surgical repair has fewer chances of rupture compared to non-surgical repair, there are chances of infection. Among open surgical repairs, end-to-end suturing has better outcomes due to fewer postoperative complications. Clinically, the history recorded in almost all patients reveals the sudden feeling of a pop sound, immediate pain, persistent weakness, balance disturbance, and gait abnormalities.

## Case presentation

A 27-year-old male laborer by occupation came to our clinic with pain over the left ankle for the last five months. History revealed trauma caused by a heavy object (metal) at his workplace five months ago. Physical examination revealed tenderness and swelling over the left heel. Plantar flexion was restricted. The patient experienced pain while walking and was unable to sit cross-legged and squat.

**Figure 1:**
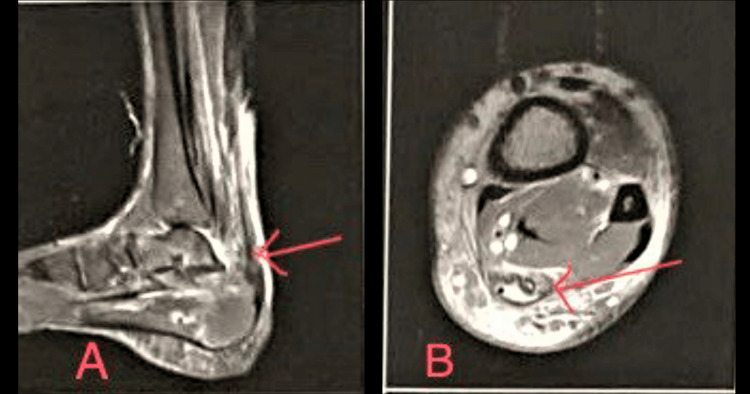
T2-weighted MRI showing hyperintense signal suggestive of Achilles tendon rupture. A: sagittal section; B: axial section.

A plain radiograph of the ankle and foot was obtained to rule out any fracture, and MRI was done to confirm the diagnosis of Achilles tendon rupture, as shown in Figure 1.

**Figure 2:**
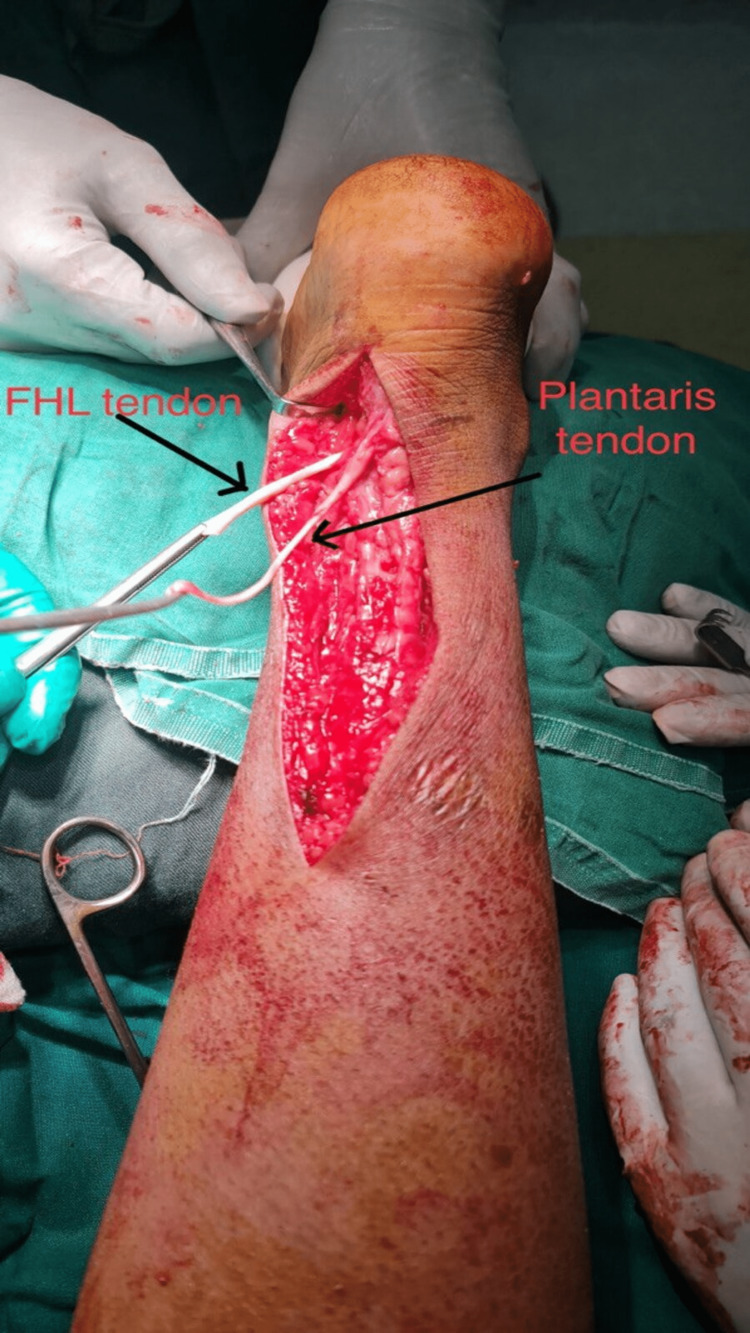
Intraoperative picture of the flexor hallucis longus and plantaris muscle tendons.

Achilles tendon repair was planned. The patient was taken to the operating room under spinal anesthesia in a prone position with a tourniquet inflated, and the Achilles tendon was exposed using a posterior approach. The fibrosis between the cut ends was cleared and a 3 cm gap was measured between the cut ends. The surgical technique used in this case involved using the flexor hallucis longus and plantaris muscle tendons to augment the cut distal portion of the Achilles tendon, as shown in Figure 2.

**Figure 3:**
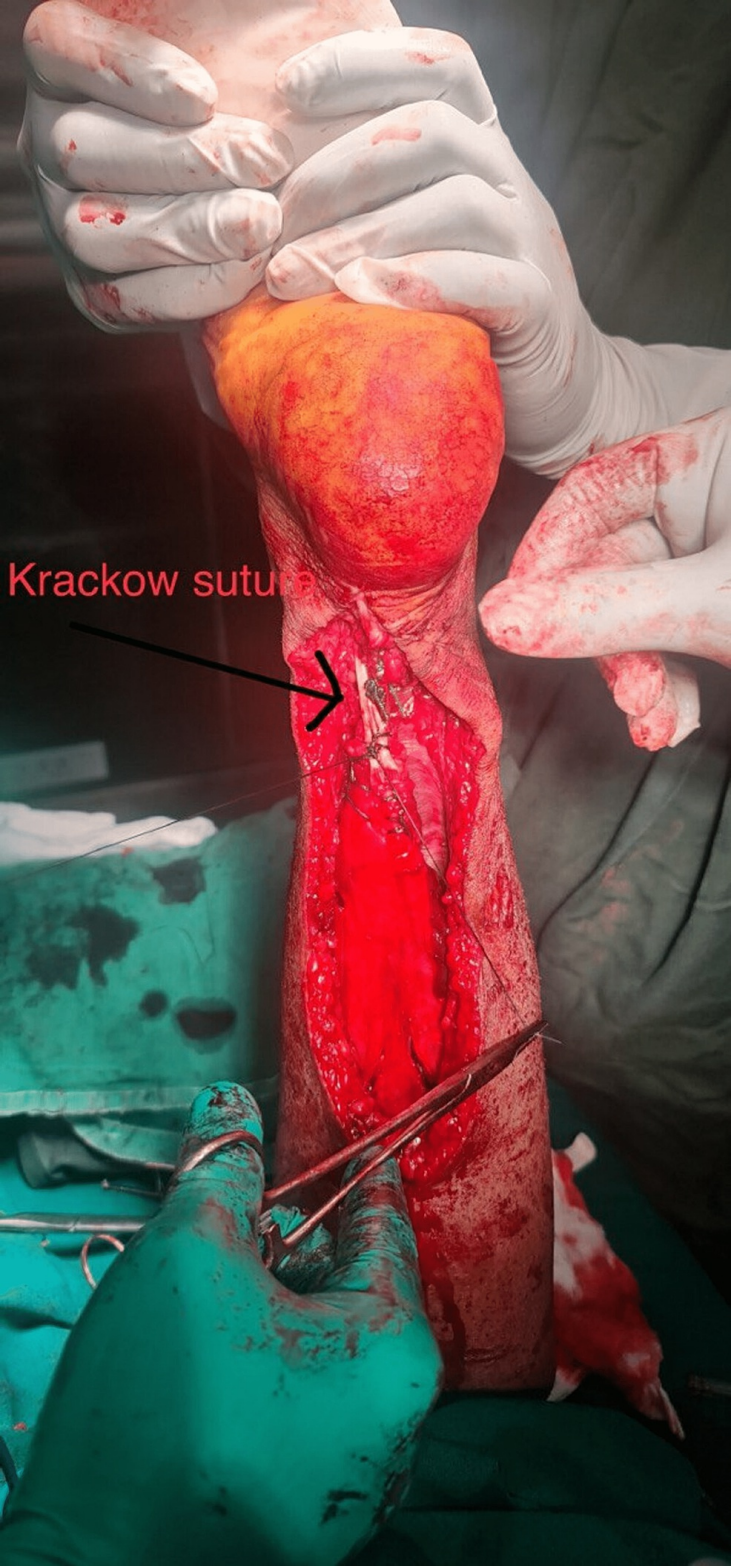
Intraoperative picture showing the Achilles tendon after Krackow suturing.

The proximal part of the Achilles tendon was lengthened using the V-Y plasty technique. The two stumps were sutured to one another using the Krackow technique, as shown in Figure 3.

**Figure 4:**
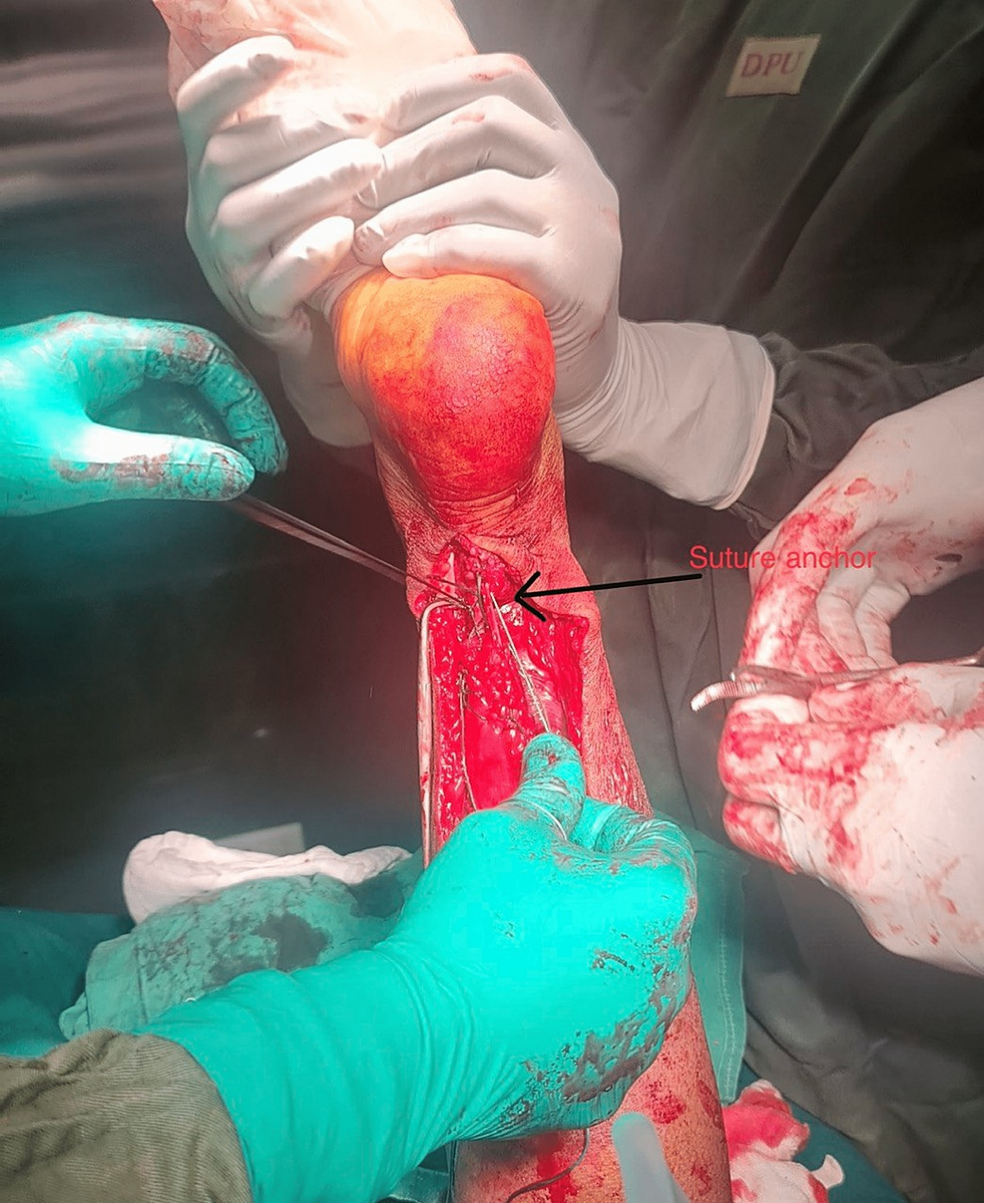
Intraoperative picture showing the reconstructed tendon with a bioabsorbable suture anchor.

Finally, the reconstructed tendon was attached to the calcaneal tuberosity using a bioabsorbable suture anchor, as shown in Figure 4.

**Figure 5:**
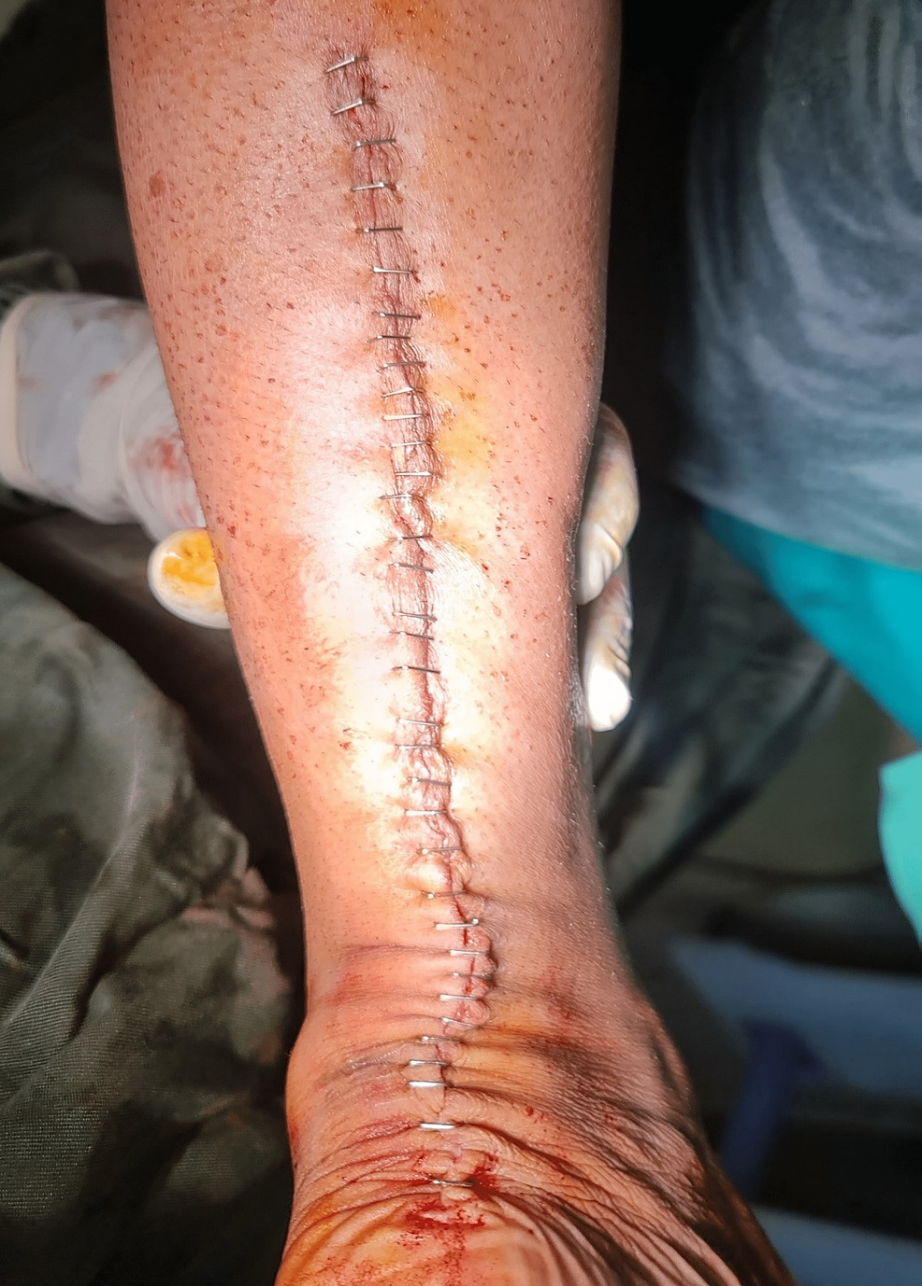
Postoperative surgical wound after closure.

Routine closure was done, as shown in Figure 5, and the postoperative period was uneventful.

The patient was given a plaster slab in plantar flexion of 30 degrees. Suture removal was done two weeks post-surgery, and the wound was found to be healed by primary intention with no gaping. After three weeks, the plantar flexion was reduced by approximately 10 degrees every week until the neutral ankle position was attained at around eight weeks post-surgery. The slab was discontinued after eight weeks, and partial weight bearing started along with active as well as passive ankle dorsiflexion exercises. Full range of motion of the ankle joint was achieved at 12 weeks. The American Orthopedic Foot and Ankle Score was used to evaluate the functional outcome of the ankle, with a score of 96 suggestive of an excellent outcome [7].

## Discussion

The best management for Achilles tendon rupture remains debatable. This case report aimed to restore the anatomy of the Achilles tendon and provide the best functional outcome to the patient using four different surgical techniques. The main aim of open repair is to maintain the length, tonicity, and anatomy of the Achilles tendon. Major complications of Achilles tendon repairs include infection, skin ulcer, sural nerve injury, re-rupture, and necrosis. The strength of primary end-to-end repair is based on suture material and technique.

The advantage of Achilles tendon repair with flexor hallucis longus transfer is early weight-bearing after the rupture. Complications include clawed hallux deformity, transfer metatarsalgia, and neurovascular injury during harvest.

Achilles tendon repair with the flexor hallucis longus tendon shows hypertrophic scar; moreover, deep vein thrombosis can also occur due to long operating time. There is a high risk of mortality and morbidity due to large incisions and more operating time. The re-rupture rate shows no significant difference between end-to-end repair and augmented repair with flexor hallucis longus [4].

If left ignored, chronic Achilles tendon rupture can be diagnosed four to six weeks after the initial injury [8]. The most frequently cited symptoms include pain, diminished strength, exhaustion, and stiff ankles. After the initial week of damage, scar tissue bridges the ruptured defect. After removing scar tissue, a palpable big gap between the rupture ends is frequently visible. Achilles tendon rupture with a wide gap is difficult to cure for the majority of orthopedic surgeons. In the therapeutic management of the Achilles tendon, numerous reconstruction techniques are employed, including tendon transfer, gastrocnemius fascial turndown flap, allograft reconstruction, autograft reconstruction, synthetic graft augmentation, and biologic matrix augmentation. In the traditional procedure, V-Y tendon plasty is an efficient and cost-effective technique. When the defect is medium and large-sized (more than 2 cm), a V-Y tendon plasty can be done. The Achilles tendon’s full strength can be recovered, enhancing patients’ degree of activity.

In the treatment of chronic Achilles tendon rupture, a gastrocnemius fascial turndown flap can also be used to close gaps larger than 2 cm. It is also a helpful method for fixing severe recurrent Achilles tendon ruptures. The gastrocnemius fascial turndown flap, however, causes considerable pain and weakness of the tendon for the majority of patients because the gastrocnemius fascial flap’s strength is lower than the strength of the tendon. The Achilles tendon tear can be treated more quickly by revascularizing the tendon of the V-Y tendon plasty from the soleus muscle. In contrast, revascularization of the soleus muscle cannot be accomplished immediately by the gastrocnemius fascial turndown flap. As a result, in our view, the rupture might heal more gradually. Performing a gastrocnemius fascial turndown flap should not be the first surgical option in cases of chronic Achilles tendon rupture.

## Conclusions

In our case report, we managed a patient with a chronic rupture of Achilles tendon using a combination of surgical techniques, including flexor hallucis longus and plantaris muscle tendon augmentation, V-Y plasty, Krackow suturing, and bioabsorbable suture anchor. The outcome was excellent as per the American Orthopedic Foot and Ankle Score without any major complications which were anticipated before surgery. The patient recovered well and the full range of motion of the ankle joint was achieved at the 12-week follow-up.
